# Radiobiological and dosimetric impact of RayStation pencil beam and Monte Carlo algorithms on intensity‐modulated proton therapy breast cancer plans

**DOI:** 10.1002/acm2.12676

**Published:** 2019-07-25

**Authors:** Suresh Rana, Kevin Greco, E. James Jebaseelan Samuel, Jaafar Bennouna

**Affiliations:** ^1^ Department of Radiation Oncology Miami Cancer Institute, Baptist Health South Florida Miami FL USA; ^2^ Department of Radiation Oncology Herbert Wertheim College of Medicine, Florida International University Miami FL USA; ^3^ Department of Physics, School of Advanced Sciences Vellore Institute of Technology (VIT) University Vellore Tamil Nadu India; ^4^ Department of Radiation Oncology Moffitt Cancer Center Tampa FL USA

**Keywords:** breast cancer, dose calculation algorithms, EUD, IMPT, Monte Carlo, NTCP, TCP

## Abstract

**Purpose:**

RayStation treatment planning system employs pencil beam (PB) and Monte Carlo (MC) algorithms for proton dose calculations. The purpose of this study is to evaluate the radiobiological and dosimetric impact of RayStation PB and MC algorithms on the intensity‐modulated proton therapy (IMPT) breast plans.

**Methods:**

The current study included ten breast cancer patients, and each patient was treated with 1–2 proton beams to the whole breast/chestwall (CW) and regional lymph nodes in 28 fractions for a total dose of 50.4 Gy relative biological effectiveness (RBE). A total clinical target volume (CTV_Total) was generated by combining individual CTVs: AxI, AxII, AxIII, CW, IMN, and SCVN. All beams in the study were treated with a range shifter (7.5 cm water equivalent thickness). For each patient, three sets of plans were generated: (a) PB optimization followed by PB dose calculation (PB‐PB), (b) PB optimization followed by MC dose calculation (PB‐MC), and (c) MC optimization followed by MC dose calculation (MC‐MC). For a given patient, each plan was robustly optimized on the CTVs with same parameters and objectives. Treatment plans were evaluated using dosimetric and radiobiological indices (equivalent uniform dose (EUD), tumor control probability (TCP), and normal tissue complication probability (NTCP)).

**Results:**

The results are averaged over ten breast cancer patients. In comparison to PB‐PB plans, PB‐MC plans showed a reduction in CTV target dose by 5.3% for D_99%_ and 4.1% for D_95%_, as well as a reduction in TCP by 1.5–2.1%. Similarly, PB overestimated the EUD of target volumes by 1.8─3.2 Gy(RBE). In contrast, MC‐MC plans achieved similar dosimetric and radiobiological (EUD and TCP) results as the ones in PB‐PB plans. A selection of one dose calculation algorithm over another did not produce any noticeable differences in the NTCP of the heart, lung, and skin.

**Conclusion:**

If MC is more accurate than PB as reported in the literature, dosimetric and radiobiological results from the current study suggest that PB overestimates the target dose, EUD, and TCP for IMPT breast cancer treatment. The overestimation of dosimetric and radiobiological results of the target volume by PB needs to be further interpreted in terms of clinical outcome.

## 1 INTRODUCTION

Intensity‐modulated proton therapy (IMPT) is used for the treatment of breast cancer at many proton centers across the world. Literature^1^, ^2^ has shown that proton therapy for breast cancer could potentially reduce normal tissue complication probability (NTCP) by reducing side effects such as cardiac and pulmonary toxicities. It is paramount that the reduction of NTCP must be accompanied by an increase in tumor control probability (TCP) to prevent tumor recurrence. Both the TCP and NTCP are calculated based on the absorbed dose in disease sites and normal tissues, respectively. Hence, the accuracy of proton dose calculation algorithm is critical in estimating the absorbed dose in tumors and organs at risk (OARs).

RayStation (version 6.1.1.2; RaySearch Laboratories, Stockholm, Sweden) employs analytical pencil beam (PB) and Monte Carlo (MC) algorithms for proton dose calculations. Several studies^3^, ^4^, ^5^, ^6^, ^7^, ^8^ have highlighted the limitation of PB algorithm within RayStation for dose calculation, especially in the presence of range shifter and inhomogeneities. For instance, Saini et al.^3^ found that MC is superior to PB when a range shifter is employed with oblique beams, large air gaps, and/or heterogeneous media. Taylor et al.^4^ demonstrated that MC calculations are more accurate than PB calculations when compared to physical measurements. Shirey et al.^6^ showed better accuracy of MC compared to PB when treatment involves the range shifter and superficial lesions.

Although superior dose prediction accuracy of MC over PB has been well established,^3^, ^4^, ^5^, ^6^, ^7^, ^8^ literature investigating the impact of RayStation PB and MC on IMPT breast cancer treatment is scarce. The investigation of proton dose calculation algorithms for breast treatment is particularly important due to the presence of a tumor at a shallower depth and range shifter in the proton beam path. The addition of range shifter at the end of the nozzle exit reduces the beam energy. This is necessary to achieve a full dose modulation of the tumor volume, but the range shifter creates an air gap between its downstream and patient surface, thus increasing in‐air spot size.^9^

Tommasino et al.^7^ included five breast cancer patients and the treatment plans were optimized with PB and recalculated with PB and MC. Additionally, Tommasino et al.^7^ performed the phantom measurements to demonstrate better accuracy using MC than using PB. Liang et al.^8^ did a more comprehensive dosimetric study by including 20 breast cancer patients. In their study,^8^ the authors used both PB and MC for plan optimization as well as dose calculations, whereas MC for plan optimization was not addressed by Tommasino et al.^7^ It is worth noting that both studies evaluated PB and MC on IMPT breast plans using dosimetric indices. However, at the time of writing this paper, the radiobiological impact of RayStation PB and MC on IMPT breast plans is yet to be investigated.

The goal of this study is twofold. First, we investigated the radiobiological impact of RayStation PB and MC algorithms on IMPT breast plans. Specifically, treatment plans are evaluated in terms of equivalent uniform dose (EUD), TCP, and NTCP. Our study included plans optimized using PB and MC as well as dose calculated using PB and MC. Second, since there is only one dosimetric study^8^ from a single institution investigating the use of RayStation MC for plan optimization of IMPT breast cancer planning, independent research from another institution on this topic is essential. Our study aims to supplement the work of Liang et al.^8^ by comparing the dosimetric results of PB and MC for IMPT breast cancer treatment. Additionally, for each case, we have presented a more comprehensive analysis of plan robustness and computational time — results of these two parameters were not provided in detail in a previous publication.^8^

## 2 MATERIALS AND METHODS

### 2.1 Patients, CT simulation, and contouring

The current study consisted of ten female left breast cancer patients who have been treated using IMPT at our center between 11/2017 and 01/2019. All ten patients received treatment to the chest wall (CW) or whole breast. For all ten patients, the treatment also included regional lymph nodes. Patients were simulated on Siemens computed tomography (CT) Scanner (Siemens Healthcare, Forcheim, Germany) in head‐first supine treatment position with arms above their heads based on our institutional protocol. This includes a vac‐lok and wing board for immobilization devices and a free breathing CT scan with a slice thickness of 2 mm.

For contouring of target volumes and OARs, CT images were transferred either to RayStation or Velocity (Varian Medical Systems, Palo Alto, CA, USA). A total clinical target volume (CTV_Total) was generated by combining individual CTV structures: breast or CW, axillary level I–III nodes (AxI‐III), internal mammary nodes (IMN), and supraclavicular nodes (SCVN). The OARs included the heart, left lung, right lung, esophagus, left anterior descending artery (LAD), and skin (either 3 mm (CW) or 5 mm (whole breast) inward from the body surface).

### 2.2 Dose prescription and treatment planning

All ten patients were treated for a total dose of 50.4 Gy relative biological effectiveness (RBE) in 28 fractions on a ProteusPLUS PBS proton therapy system^10^ (Ion Beam Applications, Louvain‐la‐Neuve, Belgium). Treatment plans were generated in RayStation (v6.1.1.2) using 1–2 beams, and each beam included the range shifter of 7.5 cm water equivalent thickness made up of lucite. A 5 mm setup uncertainty on CTV was used for the robust optimization for a total of seven scenarios. All treatment plans were robustly optimized with the goal of 95% of CTV receiving at least 95% of the prescription dose while minimizing dose to the OARs. All plans were computed with a dose calculation grid size of 3 mm. For each case, three plans were generated using identical beam angles, air gap, optimization structures, optimization constraints, and optimization settings. A sampling history of 10,000 ions/spot was used for MC optimization, and a statistical uncertainty of 0.5% was used for MC dose calculation.

1. PB‐PB Plan: The plan was optimized using PB followed by dose calculation using PB.
2. PB‐MC Plan: The plan was optimized using PB followed by dose calculation using MC.
3. MC‐MC Plan: The plan was optimized using MC followed by dose calculation using MC.

### 2.3 Dosimetric analysis

The CTV_Total was evaluated for the mean dose (D_mean_), the dose received by 99% of the volume (D_99%_), 95% of the volume (D_95%_), and 2% of the volume (D_2%_). The D_mean_ was calculated for the left anterior descending artery (LAD), heart, and esophagus, whereas the dose received by 0.03 cc (D_max_) was calculated for the skin. The ipsilateral lung (i.e., left lung) was evaluated for the relative volume that received 20 and 5 Gy(RBE) (V_20_ and V_5_, respectively), whereas the contralateral lung (i.e., right lung) was evaluated for the V_5_.

### 2.4 EUD Analysis

Equivalent uniform dose evaluation was performed using the cumulative dose volume histograms (DVHs) of the proton treatment plans (PB‐PB, PB‐MC, and MC‐MC). EUD is based on the Niemierko's phenomenological model.^11^

The EUD^11^, ^12^ is defined as.

(1) $$\text{EUD}={\left(\sum_{i=1}\left(v_{i}{\text{EQD}}_{i}^{a}\right)\right)}^{\frac{1}{a}}$$

(2) $$\text{EQD}=D\times \frac{\left(\frac{\alpha }{\beta }+\frac{D}{n_{f}}\right)}{\left(\frac{\alpha }{\beta }+2\right)}$$

In eq. (1), *a* is a unit less model parameter that is specific to the normal structure or tumor of interest, and *v_i_* is unit less and represents the *i^th^* partial volume receiving dose *D_i_* in Gy.^11^, ^12^ Since the relative volume of the whole structure of interest corresponds to 1, the sum of all partial volumes *v_i_* will equal 1^11^, ^12^ The EQD is the biologically equivalent physical dose of 2 Gy. In eq (2), n_f_ and d_f_ = D/n_f_ are the number of fractions and dose per fraction size of the treatment course, respectively. The α/β is the tissue‐specific linear‐quadratic (LQ) parameter of the organ being exposed. The EUD calculations in this study are based on the parameters listed in Table 1.^14^, ^15^, ^16^

**Table 1:** Radiobiological parameters of EUD & TCP calculations for the breast cancer plans.

Parameter	Values	Reference
D_50_ (Gy(RBE))	30.89	14, 15
γ	1.3	14, 15
α/β	4	16
a	−7.2	14, 15

EUD, equivalent uniform dose; TCP, tumor control probability; RBE, relative biological effectiveness.

### 2.5 TCP Analysis

The Poisson linear quadratic (PoissonLQ) radiobiological model^13^ employed within RayStation was used to estimate the TCP of CTV_Total, CTV_breast, CTV_AxI, CTV_AxII, CTV_AxIII, CTV_IMN, and CTV_SCVN. The TCP‐PossionLQ model is defined as^13^:

(3) $$\begin{array}{c} \text{TCP}\left(D\right)=\prod_{i=1}^{M}{\left[\exp\left(-N_{0}\exp\left(\sum_{k=1}^{n}\left\{-\alpha d_{k,i}-\beta d_{k,i}^{2}\right\}\right)\right)\right]}^{\frac{v_{i}}{V_{\mathrm{ref}}}}\\ \text{TCP}\left(D\right)=\prod_{i=1}^{M}{\left[\exp\left(-\exp\left[e\gamma -\frac{{\text{EQD}}_{2,i}}{D_{50}}\left(e\gamma -\ln(\ln(2))\right)\right]\right)\right]}^{\frac{v_{i}}{V_{\mathrm{ref}}}} \end{array}$$

where, *M*, total number of voxels; *D*, total dose; *D*_k,i_, dose to the k^th^ fraction to voxel i; *n*, total number of fractions; *N*_0_, initial number of cells; α, parameter of LQ model; *β*, parameter of LQ model; *v*_i_/*V*_ref_, relative volume of voxel i compared to the reference volume; *D*_50_, dose giving a 50% response probability; γ, maximum normalized gradient of the dose response curve; EQD_2,i_, equivalent dose in voxel i given in 2 Gy‐fractions.

The values of radiobiological parameters^14^, ^15^, ^16^ used for TCP calculations are provided in Table 1.

### 2.6 NTCP Analysis

The Lyman‐Kutcher‐Burman (LKB) model employed within RayStation^13^ was used to calculate the NTCP of the heart, lung (ipsilateral), and skin. The LKB model is defined as^13^:

(4) $$\begin{array}{c} \text{NTCP}\left(D\right)=\frac{1}{\sqrt{2\pi }}\int_{-\infty }^{t}e^{-\frac{x^{2}}{2}dx}\\ t=\frac{D_{\text{eff}}-D_{50}}{m.D_{50}} \end{array}$$

(5) $$D_{\text{eff}}=\sum_{i=1}^{M}{\left(\frac{v_{i}}{V_{\text{ref}}}{\text{EQD}}_{i}^{1/n}\right)}^{n}$$

where, *D*, total dose; *D*_50_, dose giving a 50% response probability; *m*, slope of the response curve; *M*, total number of voxels; *n*, parameter reflecting the biological properties of the organ specifying volume dependence; v_i_/*V*_ref_, relative volume of voxel i compared to the reference volume; EQD_i_, equivalent dose in voxel i given in 2 Gy‐fractions.

The values of radiobiological parameters^17^, ^18^, ^19^, ^20^ used for NTCP calculations are provided in Table 2.

**Table 2:** Radiobiological parameters of NTCP calculations for the breast cancer plans.

Structure	D_50_ (Gy(RBE))	m	n	Reference
Heart	48	0.1	0.35	17, 18
Lung (ipsilateral)	37.6	0.35	0.87	18, 19
Skin	39	0.14	0.38	20

NTCP, normal tissue complication probability; RBE, relative biological effectiveness.

### 2.7 Statistical analysis

In order to test the statistical significance of dosimetric and radiobiological results in the current study, the Mann‐Whitney U‐test was performed. A p value of less than 0.05 was considered to be statistically significant.

## 3 RESULTS

### 3.1 Dosimetric analysis

**Figure 1:**
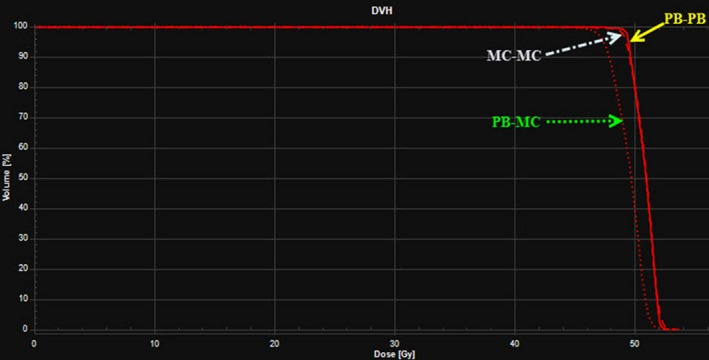
A sample dose volume histograms of clinical target volume (CTV_Total) in PB optimization followed by PB dose calculation, PB optimization followed by MC dose calculation, and MC optimization followed by MC dose calculation plans of an example patient. Treatment planning goal: CTV_Total D95% = 95% of prescription dose (50.4 Gy relative biological effectiveness (RBE)).

**Figure 2:**
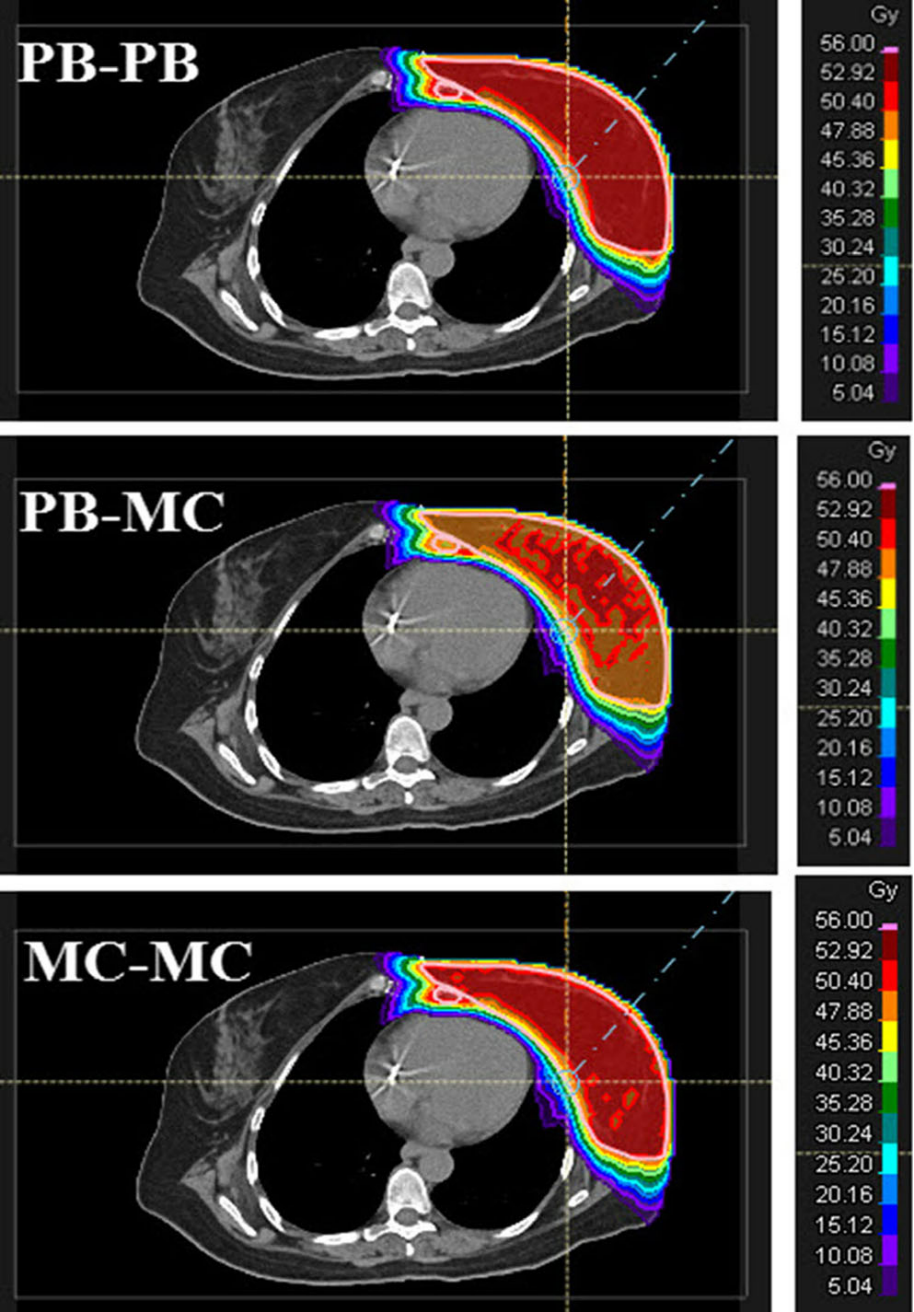
Dose distributions in PB optimization followed by PB dose calculation, PB optimization followed by MC dose calculation, and MC optimization followed by MC dose calculation plans of an example patient. Treatment planning goal: clinical target volume (CTV_Total) D95% = 95% of prescription dose (50.4 Gy relative biological effectiveness (RBE)).

Table 3 shows the dosimetric results of nominal PB‐PB, PB‐MC, and MC‐MC plans. The results are averaged over ten breast cancer patients. The recalculation of PB plans with MC showed the reduction in dose to the CTV_Total by the average differences of 5.3% for D_99%_ (*P* = 0.001), 4.1% for D_95%_ (*P* < 0.001), 2.7% for D_mean_ (*P* < 0.001), and 1% for D_2%_ (*P* = 0.112). The doses to the CTV_Total in MC‐MC and PB‐PB plans were comparable with no statistical significance (*P *> 0.05). Specifically, on average, the difference in CTV_Total dose between MC‐MC and PB‐PB plans was less than 0.5% for both D_95%_ and D_mean_ and 1.4% for D_99%_. The D_2%_ was similar in both MC‐MC and PB‐PB plans. The current study used the treatment planning goal of CTV_Total D_95%_ = 95% of the prescription dose. On average, the CTV_Total D_95%_ was 97.5%, 93.5%, and 97.1% in PB‐PB, PB‐MC, and MC‐MC plans, respectively. For PB‐PB plans, nine patients had CTV_Total D_95%_> 95% and one patient had CTV_Total D_95%_ = 94.8%. MC‐MC plans also exhibited similar results such that eight patients had CTV_Total D_95%_> 95% and two patients had CTV_Total D_95%_ = 94.6% and 94.8%. However, PB‐MC plans produced inferior results, and there was only one patient with CTV_Total D_95%_ = 95.1%, and the other nine patients had CTV_Total D_95%_ results ranging from 91.4% to 94.5%. Figure 1 shows a sample DVH of CTV_Total in PB‐PB, PB‐MC, and MC‐MC plans of an example patient. Figure 2 shows the dose distributions in PB‐PB, PB‐MC, and MC‐MC plans of an example patient.

**Table 3:** Dosimetric results in nominal PB‐PB, PB‐MC, and MC‐MC plans of breast cancer. The results are averaged over ten breast cancer patients.

		PB‐PB Avg. (range)	PB‐MC Avg. (range)	*P*‐value	MC‐MC Avg. (range)	*P*‐value
CTV_Total	D_99%_ (%)	95.5 (91.6–98.8)	90.4 (87.9–92.6)	<0.001	94.1 (92.1–98.1)	0.174
	D_95%_ (%)	97.5 (94.8–99.6)	93.5 (91.4–95.1)	<0.001	97.1 (94.6–99.3)	0.385
	D_mean_ (%)	100.3 (98.4–101.5)	97.6 (95.3–98.6)	<0.001	100 (98.2–101.2)	0.290
	D_2%_ (%)	102.6 (100.2–104.3)	101.6 (98.0–104.1)	0.112	102.7 (100.1–104.6)	0.821
Heart	D_mean_ (Gy(RBE))	0.45 (0.12–1.07)	0.47 (0.1–0.95)	0.762	0.47 (0.11–1.05)	0.970
LAD	D_mean_ (Gy(RBE))	3.37 (0.43–9.21)	3.10 (0.43–9.06)	0.764	3.64 (0.33–11.17)	0.910
Esophagus	D_mean_ (Gy(RBE))	6.02 (3.33–17.24)	5.94 (3.63–17.53)	0.705	6.28 (3.71–19.26)	0.850
Skin	D_max_ (%)	100.2 (94.6–103.1)	101.2 (95.2–105.8)	0.290	100.9 (94.8–104)	0.406
Left lung	V_20_ (%)	12.4 (2.2–22.7)	13.9 (2.6–25.3)	0.406	13.1 (4.9–24.5)	0.597
Left lung	V_5_ (%)	32.8 (21.6–47.5)	37.6 (26.7–53.4)	0.082	36.8 (31.1–51.9)	0.049
Right lung	V_5_ (%)	1.7 (0–9.4)	0.9 (0–4.1)	0.597	1.1 (0–4.2)	0.940

PB‐PB, PB optimization followed by PB dose calculation; PB‐MC, PB optimization followed by MC dose calculation; MC‐MC, MC optimization followed by MC dose calculation; CTV, clinical target volume; LAD, left anterior descending artery; RBE, relative biological effectiveness.

*P*‐value for PB‐MC vs. PB‐PB.

*P*‐value for MC‐MC vs. PB‐PB.

The average difference in D_mean_ to the heart, LAD, and esophagus among different plans (PB‐PB, PB‐MC, and MC‐MC) was less than 0.5 Gy(RBE) (*P* > 0.05). The difference in D_max_ to the skin between PB‐MC and PB‐PB plans ranged from −2.3% to 3.7% with no statistical significance (*P* = 0.290). The positive difference means PB‐MC plan has higher D_max_ than PB‐PB plan. MC‐MC plans produced consistently higher D_max_ to the skin except in one case. The difference in D_max_ to the skin between MC‐MC and PB‐PB plans ranged from −0.3% to 1.8% with no statistical significance (*P* = 0.406). For the ipsilateral lung, the average V_20_ among PB‐PB, PB‐MC, and MC‐MC plans was similar (12.4% vs. 13.9% vs. 22.3%, respectively). However, in comparison to PB‐PB plans, the difference in V_5_ of the ipsilateral lung was slightly higher in PB‐MC plans (2.7% to 8%; *P* = 0.082) and MC‐MC plans (0.7% to 13.8%; *P* = 0.049). The average V_5_ of the contralateral lung was similar among PB‐PB (1.7%), PB‐MC (0.9%), and MC‐MC (1.1%) plans (*P* > 0.05).

### 3.2 Robust analysis

**Figure 3:**
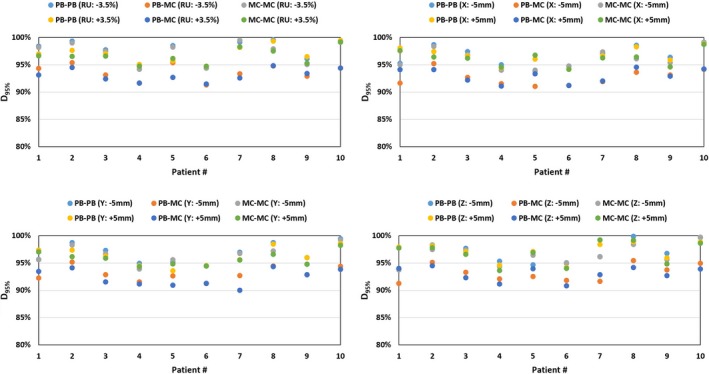
Robust evaluation of the D_95%_ of the total clinical target volume (CTV_Total) in PB‐PB (PB optimization followed by PB dose calculation), PB‐MC (PB optimization followed by MC dose calculation), and MC‐MC (MC optimization followed by MC dose calculation) plans for ten breast cancer patients. Each plan was evaluated for range uncertainty of ±3.5% and isocenter shifts of X = ±5 mm, Y = ±5 mm, and Z = ±5 mm. The acceptable robustness criteria for the intensity‐modulated proton therapy breast was 95% of the CTV_Total is covered by at least 90% of the prescribed dose (i.e., D95% ≥ 45.36 Gy(RBE)).

The robust analysis was carried out in all three sets of plans (PB‐PB, PB‐MC, and MC‐mC), and each plan was evaluated for a total of eight scenarios. Range uncertainty was evaluated for ± 3.5% and isocenter shift was evaluated for X = ±5 mm, Y = ±5 mm, and Z = ±5 mm. The acceptable robustness criteria for IMPT breast treatment was 95% of the CTV_Total is covered by at least 90% of the prescribed dose (i.e., D_95%_ ≥ 45.36 Gy(RBE)). Figure 3 illustrates the robust evaluation of the CTV_Total for all ten patients. The results showed that all three sets of plans (PB‐PB, PB‐MC, and MC‐MC) achieved the robustness criteria for the IMPT breast treatment (D_95%_ ≥ 90% of prescription dose) in all ten patients in the current study.

### 3.3 EUD analysis

**Figure 4:**
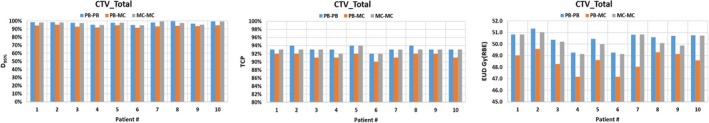
D_95%_, tumor control probability, and equivalent uniform dose of the total clinical target volume (CTV_Total) for breast cancer patients (n = 10) in PB‐PB (PB optimization followed by PB dose calculation), PB‐MC (PB optimization followed by MC dose calculation), and MC‐MC (MC optimization followed by MC dose calculation) plans generated by intensity‐modulated proton therapy (IMPT) technique.

Tables 4 and 5 show the EUD results in PB‐PB, PB‐MC, and MC‐MC plans. For all seven target volumes of each patient in the current study, a reduction in EUD (p ≤ 0.001) was noticed when PB optimized plans are calculated using MC. On average, EUD in PB‐MC plans was reduced by 2.3 Gy(RBE) for CTV_AxI, 1.8 Gy(RBE) for CTV_AxII, 1.8 Gy(RBE) for CTV_AxIII, 1.9 Gy(RBE) for CTV_CW/breast, 3.2 Gy(RBE) for CTV_IMN, and 3.1 Gy(RBE) for CTV_SCVN. The comparison between MC‐MC and PB‐PB plans showed that EUD was comparable (*P* > 0.05): 50.7 Gy(RBE) vs. 51.0 Gy(RBE) for AxI, 50.6 Gy(RBE) vs. 51.0 Gy(RBE) for AxII, 50.4 Gy(RBE) vs. 50.7Gy(RBE) for AxIII, 50.1 Gy(RBE) vs. 50.4 Gy(RBE) for CTV_CW/breast, 50.1 Gy(RBE) vs. 50.5 Gy(RBE) for CTV_IMN, and 50.2 Gy(RBE) vs. 50.5 Gy(RBE) for CTV_SCVN. EUD results of CTV_Total are displayed in Fig. 4. In comparison to PB‐PB plans, EUD of CTV_Total was comparable in MC‐MC plans (50.4 Gy(RBE) vs. 50.2 Gy(RBE); *P* = 0.430), whereas PB‐MC plans showed reduction (*P* < 0.001) in EUD of CTV_Total by an average difference of 1.9 Gy(RBE).

**Table 4:** EUD of CTV_AxI, CTV_AxII, and CTV_AxIII in nominal PB‐PB, PB‐MC, and MC‐MC plans of breast cancer.

Patient #	CTV_AxI			CTV_AxII			CTV_AxIII		
	EUD (Gy(RBE)			EUD (Gy(RBE)			EUD (Gy(RBE)		
	PB‐PB	PB‐MC	MC‐MC	PB‐PB	PB‐MC	MC‐MC	PB‐PB	PB‐MC	MC‐MC
1	51.7	49.6	51.7	51.6	50.4	51.6	50.9	50.0	50.9
2	51.8	49.9	51.4	51.7	49.8	51.3	51.4	50.0	51.1
3	50.6	48.2	50.5	50.6	48.8	50.5	50.6	49.1	50.5
4	50.5	48.9	49.5	50.5	49.3	49.5	49.9	47.3	49.7
5	51.6	49.2	51.4	51.8	50.3	51.7	51.3	49.5	50.8
6	49.9	47.7	49.8	49.8	47.4	49.8	49.9	47.3	49.7
7	50.8	47.1	50.9	50.8	48.6	50.8	50.8	48.6	50.8
8	51.2	49.0	50.3	51.2	49.4	50.3	51.2	49.6	50.2
9	51.0	49.2	50.2	50.9	49.2	50.0	50.8	49.2	49.8
10	50.8	48.6	50.8	50.7	48.8	50.7	50.7	48.6	50.7
Average	51.0	48.7	50.7	51.0	49.2	50.6	50.7	48.9	50.4
SD	0.6	0.9	0.7	0.6	0.9	0.7	0.5	1.0	0.5
*P*‐value		<0.001	0.271		<0.001	0.272		0.001	0.162

PB‐PB, PB optimization followed by PB dose calculation; PB‐MC, PB optimization followed by MC dose calculation; MC‐MC, MC optimization followed by MC dose calculation; CTV, clinical target volume, EUD, equivalent uniform dose; RBE, relative biological effectiveness.

*P*‐value for PB‐MC vs. PB‐PB.

*P*‐value for MC‐MC vs. PB‐PB.

**Table 5:** EUD of CTV_CW/Breast, CTV_IMN, and CTV_SCVN in nominal PB‐PB, PB‐MC, and MC‐MC plans of breast cancer.

Patient #	CTV_CW/Breast			CTV_IMN			CTV_SCVN		
	EUD (Gy(RBE)			EUD (Gy(RBE)			EUD (Gy(RBE)		
	PB‐PB	PB‐MC	MC‐MC	PB‐PB	PB‐MC	MC‐MC	PB‐PB	PB‐MC	MC‐MC
1	50.7	48.9	50.7	51.1	49.1	51.1	51.3	48.1	51.3
2	51.3	49.6	51.0	51.0	48.3	51.0	51.2	48.8	51.7
3	50.4	48.3	50.2	50.2	44.7	49.7	50.2	47.3	50.0
4	49.1	47.1	49.0	49.3	46.1	49.1	49.0	45.8	48.8
5	50.2	48.5	49.2	51.4	47.8	50.7	51.4	48.3	50.8
6	49.1	47.1	49.0	49.3	46.1	49.1	49.0	45.8	48.8
7	50.9	48.4	51.0	52.1	48.9	52.0	51.0	47.1	50.6
8	51.0	49.4	50.1	51.1	48.1	50.3	51.1	48.2	49.9
9	50.7	49.2	49.9	48.9	46.0	47.8	50.7	47.6	49.8
10	50.8	48.7	50.7	50.5	47.5	50.4	50.6	47.2	50.6
Average	50.4	48.5	50.1	50.5	47.3	50.1	50.5	47.4	50.2
SD	0.7	0.9	0.8	1.0	1.5	1.2	0.9	1.0	1.0
*P*‐value		<0.001	0.289		<0.001	0.447		<0.001	0.327

PB‐PB, PB optimization followed by PB dose calculation; PB‐MC, PB optimization followed by MC dose calculation; MC‐MC, MC optimization followed by MC dose calculation; CTV, clinical target volume; CW, chestwall; EUD, equivalent uniform dose; IMN, internal mammary nodes; SCVN, supraclavicular nodes; RBE, relative biological effectiveness.

*P*‐value for PB‐MC vs. PB‐PB.

*P*‐value for MC‐MC vs. PB‐PB.

### 3.4 TCP analysis

**Figure 5:**
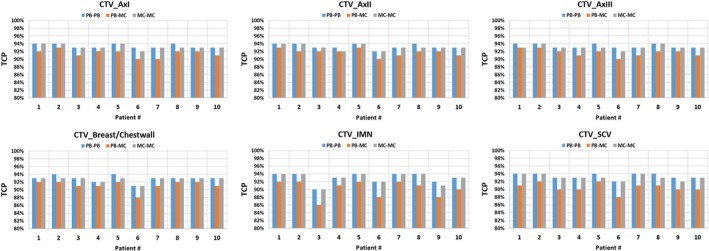
Tumor control probability of the total clinical target volumes for breast cancer patients (n = 10) in PB optimization followed by PB dose calculation, PB optimization followed by MC dose calculation, and MC optimization followed by MC dose calculation plans generated by intensity‐modulated proton therapy technique.

**Figure 6:**
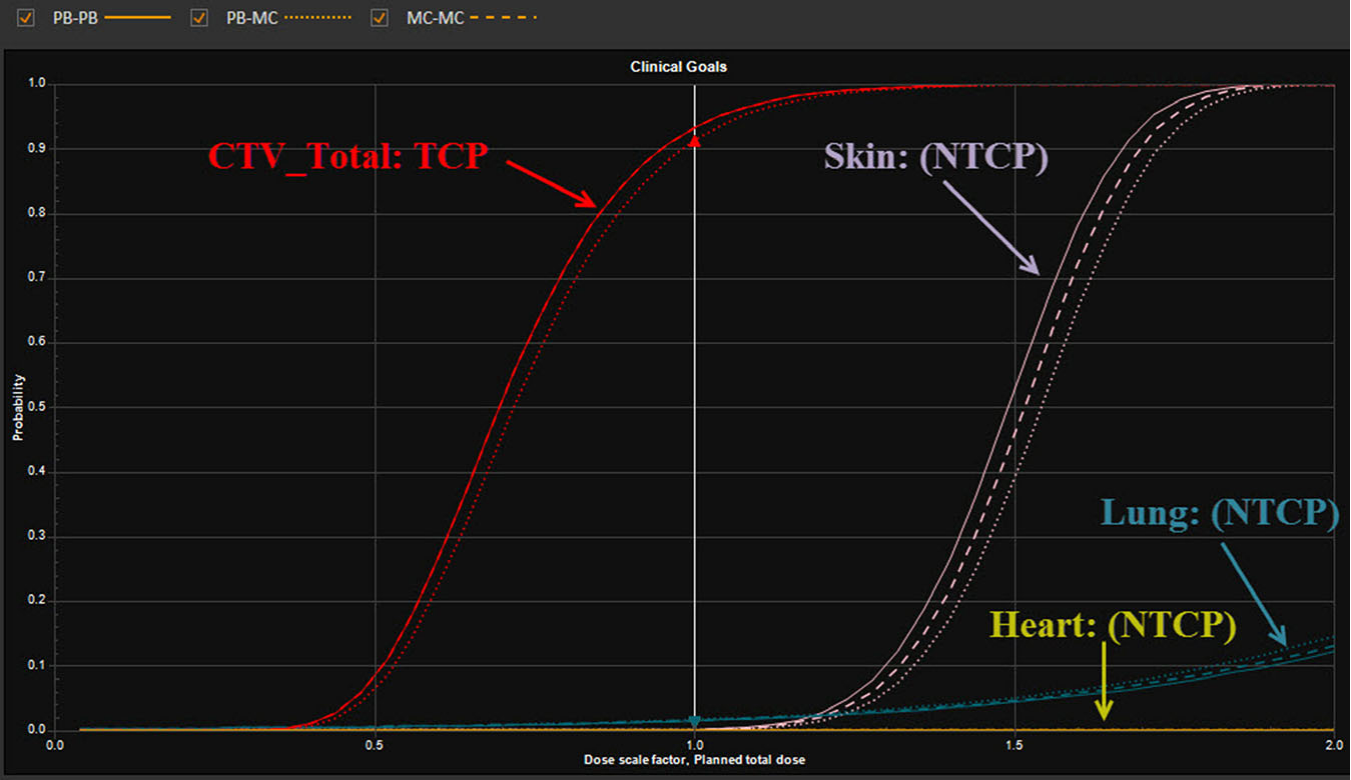
Tumor control probability clinical target volume (CTV_Total) and normal tissue complication probability (heart, skin, and left lung) in PB optimization followed by PB dose calculation, PB optimization followed by MC dose calculation, and MC optimization followed by MC dose calculation plans of an example patient.

Table 6 and Figs. 4 and 5 show the TCP results in PB‐PB, PB‐MC, and MC‐MC plans. The results are averaged over ten breast cancer patients. In comparison to PB‐PB plans, PB‐MC plans consistently showed the reduction in TCP by an average difference of 1.9% (*P* < 0.001) for CTV_AxI, 1.5% (*P* = 0.002) for CTV_AxII, 1.7% (*P* = 0.001) for CTV_AxIII, 1.7% (*P* = 0.002) for CTV_CW/breast, 2.8% (*P* = 0.003) for CTV_IMN, 2.9% (*P* < 0.001) for CTV_SCVN, and 1.8% (*P* < 0.001) for CTV_Total. In contrast, MC‐MC plans achieved TCP results similar to the ones in PB‐PB plans. On average, TCP results were similar in PB‐PB and MC‐MC plans for all CTV structures: CTV_AxI (93.4% vs. 93.2%; *P* = 0.545), CTV_AxII (93.3% vs. 93.1%; *P* = 0.571), CTV_AxIII (93.4% vs. 93.1%; *P* = 0.326), CTV_CW/breast (92.9% vs. 92.7%; *P* = 0.535), CTV_IMN (93.0% vs. 92.9%; *P* = 0.940), CTV_SCVN (93.4% vs. 93.1%; *P* = 0.385), and CTV_Total (93.2% vs. 92.9%; *P* = 0.345). Figure 6 shows the TCP of CTV_Total and in PB‐PB, PB‐MC, and MC‐MC plans of an example patient.

**Table 6:** Tumor control probability results in nominal PB‐PB, PB‐MC, and MC‐MC plans of breast cancer. The results are averaged over ten breast cancer patients.

		PB‐PB Avg. (±SD)	PB‐MC Avg. (±SD)	*P*‐value	MC‐MC Avg. (±SD)	*P*‐value
CTV_Total	TCP (%)	93.2 (±0.6)	91.4 (±0.7)	<0.001	92.9 (±0.6)	0.345
CTV_CW/Breast	TCP (%)	92.9 (±0.9)	91.2 (±1.2)	0.002	92.7 (±0.7)	0.535
CTV_AxI	TCP (%)	93.4 (±0.5)	91.5 (±1.0)	<0.001	93.2 (±0.6)	0.545
CTV_AxII	TCP (%)	93.3 (±0.7)	91.8 (±0.9)	0.002	93.1 (±0.7)	0.571
CTV_AxIII	TCP (%)	93.4 (±0.5)	91.7 (±0.9)	0.001	93.1 (±0.6)	0.326
CTV_IMN	TCP (%)	93.0 (±1.3)	90.2 (±2.1)	0.003	92.9 (±1.4)	0.940
CTV_SCVN	TCP (%)	93.4 (±0.7)	90.5 (±1.2)	<0.001	93.1 (±0.7)	0.385

PB‐PB, PB optimization followed by PB dose calculation; PB‐MC, PB optimization followed by MC dose calculation; MC‐MC, MC optimization followed by MC dose calculation; CW, chestwall; EUD, equivalent uniform dose; IMN, internal mammary nodes; SCVN, supraclavicular nodes; TCP, tumor control probability.

*P*‐value for PB‐MC vs. PB‐PB.

*P*‐value for MC‐MC vs. PB‐PB.

### 3.5 NTCP analysis

Table 7 shows the NTCP results for the heart, ipsilateral lung, and skin. There was no clear distinction among PB‐PB, PB‐MC, and MC‐MC plans in terms of NTCP results. Based on the LKB model and published radiobiological parameters used in this study, the NTCPs were 0% for the heart, ≤0.2% for the skin, and 0.4% to 1.9% for the ipsilateral (left) lung. Figure 6 shows the NTCP of the heart, left lung, and skin in PB‐PB, PB‐MC, and MC‐MC plans of an example patient.

**Table 7:** NTCP of heart, lung, and skin in nominal PB‐PB, PB‐MC, and MC‐MC plans of breast cancer.

Patient #	Heart			Skin			Ipsilateral Lung		
	NTCP (%)			NTCP (%)			NTCP (%)		
	PB‐PB	PB‐MC	MC‐MC	PB‐PB	PB‐MC	MC‐MC	PB‐PB	PB‐MC	MC‐MC
1	0.0	0.0	0.0	0.0	0.0	0.0	0.9	1.0	1.0
2	0.0	0.0	0.0	0.0	0.0	0.0	0.7	0.8	0.8
3	0.0	0.0	0.0	0.1	0.0	0.1	0.8	0.9	0.9
4	0.0	0.0	0.0	0.0	0.0	0.0	0.6	0.7	0.6
5	0.0	0.0	0.0	0.0	0.0	0.0	0.4	0.4	0.5
6	0.0	0.0	0.0	0.0	0.0	0.0	1.7	1.9	1.8
7	0.0	0.0	0.0	0.0	0.0	0.0	0.9	1.0	0.9
8	0.0	0.0	0.0	0.0	0.0	0.0	0.7	0.8	0.8
9	0.0	0.0	0.0	0.2	0.1	0.1	0.9	1.0	0.8
10	0.0	0.0	0.0	0.1	0.1	0.1	1.5	1.6	1.6
Average	0.0	0.0	0.0	0.0	0.0	0.0	0.9	1.0	1.0
SD	0.0	0.0	0.0	0.1	0.0	0.0	0.4	0.4	0.4
*P*‐value		0.968^a^	0.968^b^		0.674^a^	0.936^b^		0.384^a^	0.674^b^

PB‐PB, PB optimization followed by PB dose calculation; PB‐MC, PB optimization followed by MC dose calculation; MC‐MC, MC optimization followed by MC dose calculation; NTCP, normal tissue complication probability.

a *P*‐value for PB‐MC vs. PB‐PB.

b *P*‐value for MC‐MC vs. PB‐PB.

### 3.6 Patient‐specific quality assurance (QA) analysis

Patient‐specific QA measurement was done for PB‐PB plans of all ten patients in a water tank using DigiPhant‐PT (IBA Dosimetry, Schwarzenbruck, Germany) and MatriXX‐PT (IBA Dosimetry, Schwarzenbruck, Germany). A 2D gamma analysis was performed between the calculated and measured 2D dose distributions using patient‐specific QA module implemented within myQA software platform (IBA Dosimetry, Schwarzenbruck, Germany). For 2D gamma evaluation, we utilized 3% and 3 mm criteria and low‐dose threshold of 10%. A gamma passing rate of ≥ 90% was considered to be an acceptable level. Table 8 shows the gamma evaluation results of all ten patients. The average 2D gamma was 94.0% ± 2.9% with a minimum of 90.1% and a maximum of 98.9%.

**Table 8:** 2D gamma evaluation results from patient‐specific QA. Calculated (PB‐PB plans) and measured 2D dose distributions were compared using patient‐specific QA module implemented within the myQA software platform.

Patient #	Gamma passing rate (%)	
	Field 1	Field 2
1	95.4	92.9
2	90.6	98.9
3	93.5	98.3
4	96.0	
5	94.2	98.4
6	91.1	90.1
7	92.5	90.3
8	94.9	
9	94.2	
10	92.8	

PB‐PB, PB optimization followed by PB dose calculation; QA, quality assurance.

Gamma evaluation criteria = 3% and 3 mm; Low‐dose threshold = 10%; Accepted gamma passing rate ≥90%.

## 4 DISCUSSION

Previous studies on RayStation proton dose calculation algorithms were mostly focused on the dosimetric impact of algorithms involving either phantom^3^, ^4^, ^5^, ^6^, ^7^ or disease sites.^5^, ^7^, ^8^ To our best knowledge, at the time of writing this paper, there is currently no literature that has addressed the radiobiological impact of RayStation PB and MC in IMPT breast treatment plans. It is essential to investigate how dosimetric accuracy of dose calculation algorithms can be translated to the radiobiological differences in clinical patient cases. Hence, the current study was undertaken to demonstrate the radiobiological impact of RayStation PB and MC in terms of EUD, TCP, and NTCP in IMPT breast cancer treatment plans.

For breast cancer treatment, a tumor volume is often situated at a shallower depth. This necessitates the use of a proton beam with a smaller range. However, our proton system has a minimum of 4.0 cm range in water. Hence, for the treatment of shallower target such as in the case of CW/breast, a range shifter is typically used to reduce the energy of the proton beam. Although the use of range shifter allows to achieve full dose modulation of the tumor volume that may be extended close to the skin, an accurate modeling of algorithms accounting for angular distribution of a pencil proton beam after traversing the range shifter and translation of angular distribution into a geometric spread of proton beam's cross‐section at the detector/patient surfaces is critical.^9^, ^21^

It has been reported that recalculation of PB plans using MC will result in decrease in target dose and coverage. In a breast study by Tommasino et al.,^7^ the MC recomputed average dose to the planning target volume (PTV) was 7.1% lower than the prescription dose of 50 Gy. Liang et al.^8^ reported the reduction of CTV D_mean_ by 2.1% of the prescription dose in MC recomputed plans compared with PB plans. In the current study, we observed the reduction of the CTV_Total D_mean_ in PB‐MC plans by an average difference of 2.7% (range, 1.6–3.5%) when compared to PB‐PB plans. Liang et al.^8^ also reported the reduction of CTV D_99%_ and CTV D_95%_ by 3.7% and 3.4%, respectively, when PB plans are recalculated with MC. The findings from the current study agree with that of Liang et al.^8^ such that we noticed the reduction of the CTV_Total D_99%_ and D_95%_ in PB‐MC plans by an average difference of 5.3% and 4.1%, respectively, when compared to PB‐PB plans.

RayStation has made MC available for plan optimization. MC‐optimized plans offered optimal CTV coverage and dose distribution similar to the ones in the PB plan. The current study agrees with the results from the Liang et al.^8^ such that CTV_Total dose between MC‐MC and PB‐PB plans was found to be minimal (<0.5% for both D_95%_ and Dmean; 1.4% for D_99%_). The differences between Liang et al.^8^ and our study may be attributed to the planning techniques. For instance, Liang et al.^8^ normalized PB optimized plans and MC optimized plans such that 95% of the PTV was covered by 95% of the prescription dose, whereas plan normalization technique was not utilized in our study.

If MC is more accurate than PB for IMPT treatment of shallower targets requiring range shifter as reported in the literature,^3^, ^4^, ^5^, ^6^, ^7^ dosimetric results comparing PB‐PB vs. PB‐MC plans in the current study suggest that PB overestimates the target dose and coverage. Based on the radiobiological results in the current study, PB slightly overestimated the TCP for all CTV structures by 1–3% for AxI, 1–2% for AxII, 1–3% for AxIII, 1–3% for CW/breast, 2–4% for IMN, 2–4% for SCVN, and 1–2% for CTV_Total. Similarly, PB overestimated the EUD of target volumes by 1.8–3.2 Gy(RBE). However, the choice of optimization and dose calculation algorithms did not produce any noticeable differences in the NTCP of the heart, lung, and skin. It has been reported that breast cancer patients treated with proton therapy could have the risk of acute skin toxicities.^22^, ^23^ The NTCP of skin for the clinical endpoint of severe acute toxicity in our study was ≤0.2% for all ten patients. This was calculated using the LKB model and radiobiological parameters predicted by Pastore et al.^20^ It must be noted that radiobiological evaluation in our study was carried out based on radiobiological parameters that are derived from the conventional mega‐voltage X‐ray (photon) therapy. This is a limitation of our study. As more breast cancer patients are being treated using proton therapy and enrolled in clinical trials, there is a need for proton derived NTCP models correlating to the tissue toxicities of breast cancer patients. Due to lack of proton derived radiobiological parameters, researchers continue to use photon‐derived NTCP models for proton therapy.^12^, ^14^, ^25^ Recently, Blanchard et al.^24^ validated photon‐derived NTCP models that can be used to select head and neck patients for proton treatment.

The current study assumed constant RBE value of 1.1. Several publications^26^, ^27^ have demonstrated the existence of variable RBE for proton therapy and depend on the cell type, endpoint, LET, radiation dose, etc. The variability in RBE could lead to different α/β values, thus impacting EUD, TCP, and NTCP.^28^ In this study, we did not explore the impact of variable RBE on IMPT breast plans. Our future work will investigate how the combination of variable RBE and proton dose calculation algorithm can affect the radiobiological results.

**Figure 7:**
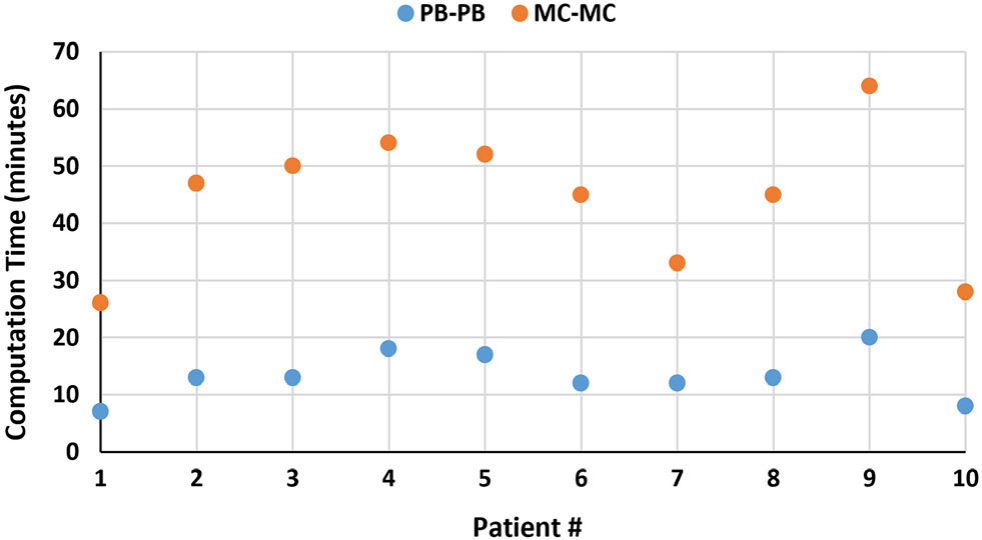
Computation time in minutes for intensity‐modulated proton therapy breast plans (PB optimization followed by PB dose calculation and MC optimization followed by MC dose calculation) of ten breast cancer patients.

One of the challenges associated with MC plan optimization is the treatment planning efficiency. Figure 7 illustrates the computation time in minutes for PB‐PB and MC‐MC plans of all ten patients. For PB‐PB plans, the average computation time was 13.3 ± 4.1 min (range, 7–20 min), whereas the average computation time for MC‐MC plans was 44.4 ± 12.1 min (range, 26–64 min). Overall, PP‐PB plans had higher computation efficiency, with an average factor of 3.4 when compared to MC‐MC plans. It must be noted that IMPT plan optimization time is dependent on several factors such as computing hardware and software resources, robustness scenarios, number of optimization structures and their constraints, and optimization settings (number of iterations and sampling history – number of ions/spot).

## 5 CONCLUSION

If RayStation MC is more accurate than PB as reported in the literature, dosimetric and radiobiological results from the current study suggest that PB overestimates the target dose, EUD, and TCP for IMPT breast cancer treatment. The overestimation of dosimetric and radiobiological results of the target volume by PB needs to be further interpreted in terms of clinical outcome. The use of RayStation MC for both plan optimization and dose calculation of IMPT breast cancer plans can provide optimal target coverage and radiobiological results (EUD and TCP for target volumes) with better accuracy.

## CONFLICT OF INTEREST

No conflicts of interest.
